# Field evaluation of a recombinase polymerase amplification assay for the diagnosis of *Schistosoma japonicum* infection in Hunan province of China

**DOI:** 10.1186/s12879-017-2182-6

**Published:** 2017-02-21

**Authors:** Weiwei Xing, Xinling Yu, Jingtao Feng, Kui Sun, Wenliang Fu, Yuanyuan Wang, Minji Zou, Wenrong Xia, Zhihong Luo, Hongbin He, Yuesheng Li, Donggang Xu

**Affiliations:** 1 The Laboratory of genomic engineering, Beijing Institute of Basic Medical Sciences, Beijing, China; 2 The key laboratory of Immune and Control of Schistosomiasis, Hunan Institute of Parasitic Diseases, Hunan, China; 3 Queensland Institute of Medical Research, Brisbane, Australia

**Keywords:** *Schistosoma japonicum*, Recombinase polymerase amplification, Field evaluation, Diagnosis

## Abstract

**Background:**

Current diagnostic methods for *Schistosoma japonicum* infection are insensitive for low-density infections. Therefore, a new diagnostic assay based on recombinase polymerase amplification (RPA) technology was established and assessed for field applification.

**Methods:**

The *S.japonicum* RPA assay was developed to target highly repetitive retrotransposon SjR2 gene of *S japonicum*, and its sensitivity and specificity were assessed by serial dilution of *S. japonicum* genomic DNA and other related worm genomic DNA respectively. The RPA diagnostic validity was first evaluated in 60 fecal samples from healthy people and patients, and then compared with other diagnostic tests in 200 high-risk individuals living in endemic areas.

**Results:**

The real time RPA assay could detect 0.9 fg *S. japonicum* DNA within 15 min and distinguish *S. japonicum* from other worms. The validity analysis of RPA for the detection of *S. japonicum* in stool samples from 30 *S. japonicum*-infected patients and 30 healthy persons indicated 100% sensitivity and specificity. When testing 200 fecal or serum samples from a high-risk population, the percentage sensitivity of RPA was 100%, whereas that of indirect hemagglutination assay (IHA) and enzyme-linked immunosorbent assay (ELISA) were 80.3% and 85.2% respectively. In addition, the RPA presented better consistency with the stool-based tests than IHA and ELISA. Overall, the RPA was superior to other detection methods with respect to detection time, sensitivity, and convenience.

**Conclusions:**

This is the first time we applied the RPA technology to the field evaluation of *S. japonicum* infection. And the results suggest that RPA-based assays can be used as a promising point-of-care test for the diagnosis of schistosomiasis.

## Background

*Schistosomiasis japonica* is a major tropical disease in China, with a >2100-year documented history [1]. With the implementation of the National Control Program supported by the Chinese government, China has made great progress in reducing *S. japonicum* infections in humans [2–5]. Today, the prevalence is becoming more and more low in most of the endemic areas. Under such circumstances, current diagnostic methods became less sensitive and specific which make the control program in a difficult situation. Generally, *S. japonicum* infections are diagnosed by direct parasitological methods or immunological methods. Parasitological detections, including the Kato-Katz (KK) thick smear and the miracidium hatching test (MHT), were regarded as golden standard for the diagnosis of schistosomiasis. However, parasitological detection is labor-intensive, time-consuming, and exhibits low sensitivity, which is not suitable for large-scale disease surveillance [6, 7]. Immunological methods include indirect hemagglutination assay (IHA) and enzyme-linked immunosorbent assay (ELISA). Both of them are more sensitive and convenient than parasitological methods. However, the above immunologic detection methods are usually not species-specific and can not discriminate between the active and past *S. japonicum* infection. Many studies demonstrated that false-positive rates of IHA and ELISA were very high in field settings [8–11]. Recently, Pan et al. verified a potential protein marker, SjSP-13, using genome-wide methods, and the SjSP-13-based ELISA kit showing 90.4% sensitivity and 98.9% specificity in a field study. However, its validity still needs further confirmation in large-scale population studies [12]. Given that the currently available diagnostic methods are not very satisfactory, development and evaluation of new strategies and tools for the control of schistosomiasis were recommended by the World Health Organization [13]. With the development of nucleic amplification technology, polymerase chain reaction (PCR) and other isothermal amplification technologies have been described for the diagnosis of schistosomiasis [14, 15]. Although PCR-based assays provide sensitive, specific and reliable tools, they are not widely utilized due to the dependence on expensive apparatus and training operator, which limits their large-scale application for clinical diagnosis [16].

In 2006, Piepenburg et al introduced a novel isothermal technology called recombinase polymerase amplification (RPA) for molecular diagnosis [17]. Unlike many other amplification methods, RPA does not require thermal denaturation of template but utilizes recombinase enzyme with opposing oligonucleotide primers to scan duplex DNA and facilitate strand exchange at cognate sites. The reaction progresses rapidly and results in specific DNA amplification from just a few target copies to detectable levels typically at temperatures between 25 °C and 42 °C. With RPA probes which contain a specific abasic nucleotide analogue flanked by a dT-fluorophore and a corresponding dT-quencher group, we can monitor amplication events in the reaction [17]. Since RPA has advantages, including a broad range of incubation temperatures (25–42 °C), shorter reaction times (typically <15 min), and more flexibility in basic laboratory settings in the field, it has gained further attention in point-of-care testing.

Here, we developed a real-time RPA assay for rapid detection of *S. japonicum* DNA in fecal samples and compared this assay with current methods in terms of sensitivity and specificity for the diagnosis of *S. japonicum* infection in high-risk populations.

## Methods

### RPA primer and probe

The highly repetitive retrotransposon SjR2 of *S. japonicum* (GenBank accession No. AF412221) was used for DNA detection as a target sequence [18–20]. The SjR2-specific primers and probe for RPA were designed according to Piepenburg [17], and the optimal combination of primers and probe were shown in Table 1. All oligonucleotides were produced by Sangon Biotech, Beijing, China.

**Table 1:** RPA primers and probe designed in this study

Name	Sequence
S. *japonicu*m RPA FP	CCAAGTCTCAGTGAAGTTGTGAAGGCTAT
S. *japonicu*m RPA RP	GTTAGTGTTCGAGACCAGTCAGATGGGATT
S. *japonicu*m RPA P	CTTAAAGCGAGGGAGAGCGGCAGGACCAGA (dT-FAM)G(THF)A(dT-BHQ1)TGACCCCTGAGATAT[3’-block]

*FP* forward primer, *RP* reverse primers, *P* probe, *dT-FAM* thymidine nucleotide carrying fluorescein, *THF* tetrahydrofuran spacer, *dT-BHQ1* thymidine nucleotide carrying black hole quencher

### DNA extraction

Samples of genomic DNA were extracted from the adult worms of *S. japonicum, S. haematobium, S. mansoni,* and *S. sinensium* by a DNeasy Tissue Kit (Qiagen, CA, USA) following the manufacturer’s instruction. The concentration and purity of the DNA were determined spectrophotometrically by readings at wavelengths of 260 nm and 280 nm (Eppendorf BioSpectrometer, Hamburg, Germany).

### RPA reactions

RPA was performed in a total volume of 50 μL, using a TwistAmp Exo kit (TwistDX Ltd., Cambridge, UK). Each reaction contained 29.5 μl TwistAmp rehydration buffer, 2.1 μl each RPA primer (210nM), 0.6 μl RPA probe (120nM), 12.2 μl nuclease-free water, 1 μl template and 2.5 μl magnesium acetate. All reagents except for the magnesium acetate and template DNA were pipette into a 0.2 ml reaction tube which contains a dried enzyme pellet. To start the reaction magnesium acetate and template DNA were added. Then the tube was placed in the Twista^TM^ incubator block (39 °C) and the fluorescence measurement was initiated to minitor the progression of RPA reactions. The preincubation was performed for 1 min and followed by incubation for 20 min, with brief mixing of the mixtures after a 4 min incubation step. In combination with the nuclease sensitive fluorophore/ quencher probes, a real-time DNA detection system was constituted. The labeled amplicon that generated in the reaction can be measured via 6-carboxyfluorescein (FAM) fluorescence using the Twista™ reader every 20 s. The RPA fluorescence data were assessed by taking a baseline relative fluorescence unit measurement.

### Real-time PCR

Real-time PCR was performed using SYBR Green Mastermix (Fermentas, Ontario, Canada) and the ABI 7500 QPCR system according to manufacturer instructions. All samples were processed in triplicate. Primers used in amplifications were as follows: SJR2: 5′-GAC AGG TTC TGG AAC ATA GG-3′; SJR2: 5′-GGT CAA TTC CGA AGA CAA TC3′.

### Sensitivity and specificity tests

The sensitivity of the RPA-assay was evaluated using five 10-fold serial dilutions ranging from 9 pg/μL to 0.9 fg/μL genomic DNA extracted from an *S. japonicum* worm. The specificity of detection was performed using genomic DNA from *S. japonicum, S. haematobium, S. mansoni,* and *S. sinensium*.

### Analysis of RPA diagnostic validity

Thirty infected fecal samples collected from Xiangyue Hospital in Yueyang city were diagnosed by the KK method as egg-positive. The other thirty uninfected fecal samples were collected from health volunteers living in the Haidian District of Beijing, where schistosomiasis is not endemic. We then used RPA technology to analyze the fecal samples and assess the sensitivity and specificity of the RPA system.

### Field evaluation

Two-hundred fishermen living in Yueyang County near Dongting Lake in the northeastern region of Hunan Province were enrolled in the field study to examine the potential diagnostic value. The inhabitants became infected with *S. japonicum* owing to frequent agricultural activities and fishing in snail-infested marshlands.. Total DNA from 500 mg of fecal sample of each participant was extracted using the QIAamp DNA Stool Mini Kit (Qiagen GmbH, Hilden, Germany) according to manufacturer instructions. The other fecal sample from each participant was analyzed with MHT and KK. 3 mL blood sample was collected from each participant and was centrifuged as 760 × g at 4 °C for 10 min. The obtained serum samples were stored as -80 °C for ELISA and IHA measurement.

### MHT and KK thick smear

The MHT was performed by adding about 30 g of feces into a metal container with a coarse wire mesh (150 holes per square inch). With the flowing water and a stirring stick, fine material was flushed into a 300-mesh nylon bag and flushed with water until the sediment was clear. Then the sediment was collected and transferred to a triangular flask containing 300 mL non-chlorinated water from the Yangtze River. Finally, the flasks were left in a well-lit room with the temperature set at approximately 28 °C and examined with a magnifying glass to check the present of swimming miracidia after 4, 6, 8, and 24 h.

The KK thick smear was performed according to a standardized methodology [21]. The method based on three slides by using fresh stool specimen, nylon screens and plastic templates (Zhejiang Ningbo Medical Instrument Factory, Ningbo, China). The three slides (41.7 mg each) were prepared from one stool sample and examined within 1 week. The intensity of infection was calculated by multiplying the number of eggs per glass slide by 24.

### ELISA and IHA

*S. japonicum* IgG ELISA kit (BIOCBD, Shenzhen, China) was used to detect the infection status of the high-risk population. All procedures were conducted according to manufacturer instructions. The IHA detection kit was purchased from Anhui Provincial Institute of Parasitic Diseases (Wuhu, China). The testing procedure was performed as described by Zhou et al [8]. The test result was recognized to be positive when a positive reaction appeared at an antibody titer of ≥1:10.

### Statistical analysis

In the field survey, the combination of the KK method and the MHT was considered the diagnostic gold standard, and the diagnostic performance of RPA was assessed by calculating sensitivity, specificity, positive predictive value (PPV), and negative predictive value (NPV). The indexes were calculated using SAS 9.2 software (SAS Institute, Inc., Cary, NC, USA), and agreement statistics were determined (JMP v9, SAS Institute Inc.) to establish the agreement between two diagnostic tests. A *p* < 0.05 was considered significant.

## Results

### Sensitivity and specificity of the RPA assay for *S. japonicum*

**Fig 1:**
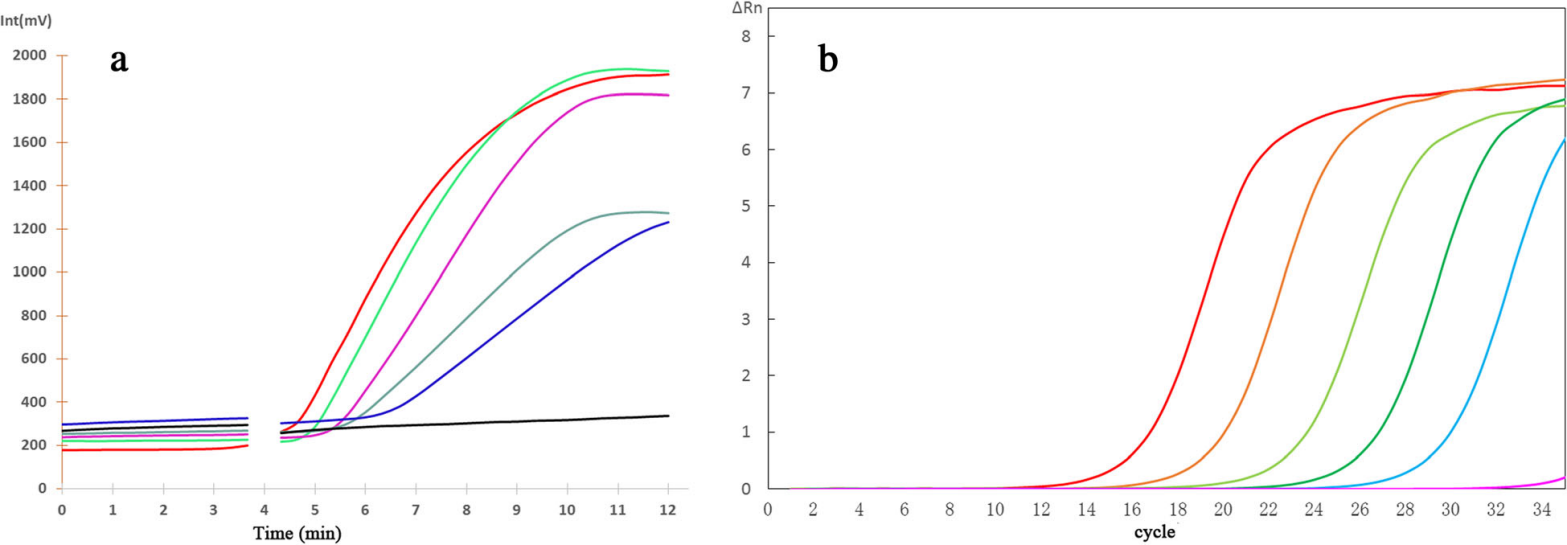
Analytical sensitivity of real-time RPA and real-time PCR for *Schistosoma japonicum* detection. Fluorescence development via real-time detection using a dilution range of 9 pg/μL–0.9 fg/μL of the *S. japonicum* genomic DNA. **a** Real-time RPA: 9 pg/μL, represented by the red line; 900 fg/μL, green; 90 fg/μL, pink; 9 fg/μL, cyan; 0.9 fg/μL, blue; negative control, black. **b** Real-time PCR: 9 pg/μL represented by the red line; 900 fg/μL, orange; 90 fg/μL, light green; 9 fg/μL, green; 0.9 fg/μL, cyan; negative control, pink

**Fig 2:**
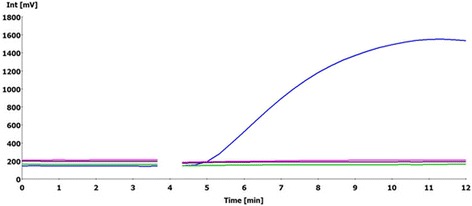
The specificity of *Schistosoma japonicum* RPA. *S. japonicum* is represented by the blue line; *S. mansoni*, black; *S. sinensium*, green; *S. haematobium*, pink

The analytical sensitivity of the assay was evaluated with 10-fold serial dilutions ranging from 9 pg/μL to 0.9 fg/μL of genomic DNA extracted from an *S. japonicum* worm. The results showed that the RPA limit of detection was 0.9 fg DNA per reaction, which is equal to that of real-time PCR (Fig. 1a and b). Although the RPA sensitivity was equal to real-time PCR, the former was far superior to the latter in detection time and convenience. RPA specificity for *S. japonicum* was confirmed by cross-detection assays using the genomic DNA of *S. mansoni, S. sinensium,* and *S. haematobium*, while none of these was amplified (Fig. 2).

### Analysis of RPA diagnostic validity

We analyzed RPA diagnostic validity using 30 infected and 30 uninfected fecal samples. Compared to KK method, RPA diagnostic sensitivity and specificity were both 100% for stool samples (Table 2). This result indicated that the RPA method was valid for *S. japonicum* diagnosis.

**Table 2:** Analysis of diagnostic validity of RPA

Subjects	Investigated	RPA-positive
Controls (negative by KK method)	30	0 (0%)
Cases (positive by KK method)	30	30 (100%)

### Comparison of the RPA assay, ELISA, IHA, MHT and the modified KK method in terms of sensitivity and specificity for the detection of *S. japonicum* infection in the field study

**Fig 3:**
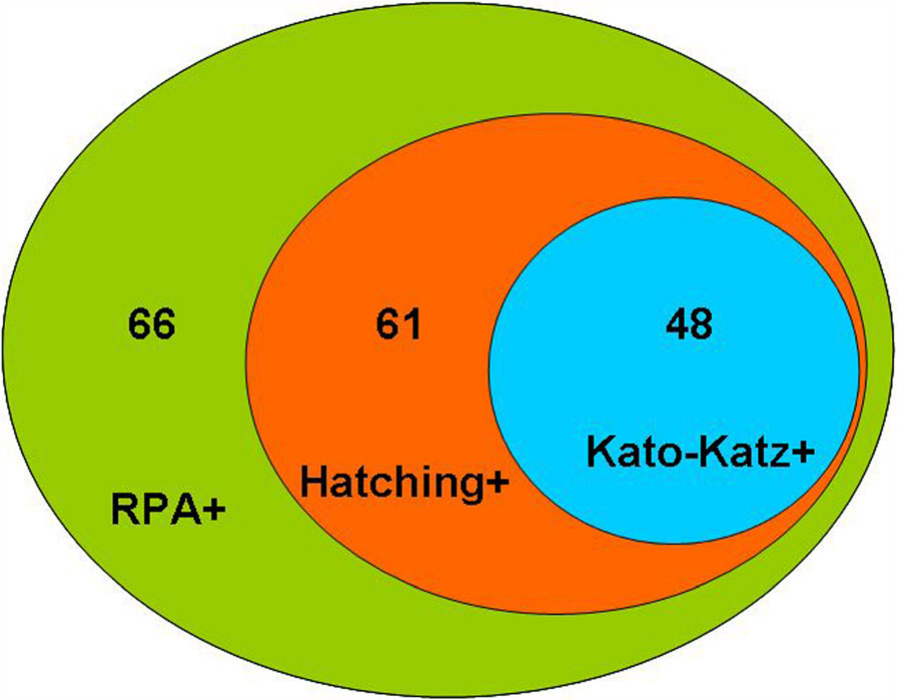
Agreement between RPA, Kato-Katz thick smear and MHT for stool-based diagnosis of *Schistosoma japonicum* infections. Values indicate the number of positive samples in each group

To further assess the RPA assay, we performed a field study to evaluate its sensitivity and specificity. The RPA diagnostic validity was compared with that of ELISA, IHA, the MHT and the modified KK method. Two-hundred inhabitants were enrolled in the study, including 92 male and 108 female participants. Among these, 164 (82%) were aged 15–54 years, and 36 (18%) were aged 55–78 years, and the local fishermen were assessed between March 2013 and April 2014. Of the 200 enrolled residents, 61 (31.5%) individuals were identified as being positive using the MHT, which included 48 (24%) individuals tested positive using the KK method. All individuals diagnosed as positive for parasitological infection were also diagnosed as being positive by RPA detection (Fig. 3). In conclusion, the RPA identified more positive cases than either, including samples negative by the other techniques (Table 3). Here, the combination of the KK and MHT methods was considered the diagnostic gold standard. The sensitivity and specificity of ELISA and IHA were 85.2% and 93.5%, 80.3% and 83.4% respectively, while RPA sensitivity and specificity was 100% and 96.4%, respectively (Table 3). In addition, the RPA showed a significantly higher degree of agreement with the gold standards (kappa: 0.942; 95% confidence interval (CI): 0.89–0.99; *p* < 0.001) relative to IHA and ELISA [IHA kappa: 0.61 (95% CI: 0.49–0.72), *p* < 0.001; ELISA kappa: 0.78 (95% CI: 0.69–0.88), *p* < 0.001] (Table 4).

**Table 3:** Estimation of diagnostic validity of RPA, IHA and ELISA

Methods	TRUE positive	FALSE negative	TRUE negative	FALSE positive	Sensitivity (%, 95% CI^a^)	Specificity (%, 95% CI^a^)	PPV% (95% CI^a^)	NPV% (95% CI^a^)
RPA	61	0	134	5	100% (100)	96.40% (99.32–99.54)	92.41% (86.06–98.83)	100% (100)
IHA	49	12	116	23	80.32% (70.37-90.45)	83.45% (77.22-89.67)	68.06% (57.37-78.82)	90.67% (85.64-95.72)
ELISA	52	9	130	9	85.24% (76.34-94.15)	93.52% (89.44-97.61)	85.37% (76.34-94.18)	93.51% (89.45-97.67)

^a^ Exact 95% confidence intervals (CIs)

**Table 4:** Agreement statistics were calculated for RPA, IHA and ELISA with stool-based tests

Diagnostic test	Degree of agreement - Kappa value	95% CI^a^	*P* value
RPA vs. golden standard	0.95	89– 99	<0.001
IHA vs. golden standard	0.61	49– 72	<0.001
ELISA vs. golden standard	0.78	69– 88	<0.001

^a^ Exact 95% confidence intervals (CIs)

## Discussion

The global schistosomiasis control program has made tremendous progress in reducing the prevalence of the disease and morbidity in many endemic areas, particularly in China [22]. However, the complete elimination of schistosomiasis associated with *S. japonicum* and the prevention of its reemergence remain difficult due to the limitations of detection methods [23]. Therefore, it is necessary to develop an efficient and convenient detection method for overall control of schistosomiasis infection.

Recently, related DNA-amplification technologies, including PCR, real-time PCR, and isothermal nucleic acid amplification, were developed to detect *S. japonicum* infections and showed potential as highly sensitive and specific techniques for the detection of parasite DNA in feces or sera, especially in regions with low-intensity infections. However, PCR and real-time PCR were extremely dependent on a well-equipped laboratory, given that testing times usually take 60–90 min, and the equipment is heavy, expensive, complex, and must be operated by qualified staff. Additionally, the drawbacks of isothermal amplification methods such as LAMP are as follows: the design of appropriate LAMP primers is more complicated than that of PCR and RPA primers, and LAMP requires higher incubation temperatures ranging from 55 °C to 65 °C for 60–75 min, with the amplification products difficult to quantify [24].

In this study, RPA technology was used to detect *S. japonicum* infection, with the diagnostic method revealing substantial advantages over current methods. First, RPA showed adequate sensitivity and specificity required for identification of low-intensity infections. The RPA limit of detection reached 0.9 fg per reaction, which was equal to real-time PCR. The non-long terminal repeat (LTR) retrotransposons (SjR2) was chose as detection targets, which were highly conserved and repeatable in the *S. japonicum* genome [20]. And we found there was no cross-reactivity with other fluke infections. Second, the RPA technique combined with the fluorescent probe achieved detection within 10–15 min, which was a much shorter duration relative to that of current methods. Third, the RPA can be operated at a relative low and constant temperature and the only requirements are primers, the probe, and the sample, while others can be stored in lyophilized form, which facilitates field testing or point-of-care applications. The above advantages make RPA more suitable for the application in field study or point of care test. Recently a LFD-RPA assay which combine recombinase polymerase amplification and lateral flow dipstick was also developed. It adopted a lateral flow system to readout the results with naked eyes which seemed really rapid and applicable in field. Nevertheless, current LFD-RPA assay requires additional steps to transfer the amplified product to another opened tube for lateral flow detection, which may lead to the possibility of nucleic acid contamination [25, 26]. Therefore, the LFD-RPA assay still needs to be further improved for practical application.

Further, the validity analysis for RPA in the detection of *S. japonicum* in the stool samples from 30 controls and 30 cases indicated that the method exhibited excellent sensitivity and specificity. Therefore, RPA technology was then applied in the field survey of *S. japonica* infection and compared with current detection methods. The field study showed that the RPA assay identified 66 enrolled participants as positive, which included all individuals diagnosed as positive by the MHT and KK methods. Among all the participants, RPA technique identified 33% of infection intensity, while the KK method only identified only 24% of infection intensity. Furthermore, when the combination of the KK method and the MHT was regarded as the diagnostic golden standard,RPA sensitivity and specificity was 100% and 96.4%, respectively, which was significantly superior to that of the IHA and ELISA methods. However, a significant drawback of RPA technology is its high cost per reaction. The primers and RPA exo reaction kits together cost approximately $4.3, which was higher than current detection technology. Of course, with availability and throughput increase, the prices are likely to decrease in the future. Besides, some more simple and cheap DNA extraction methods are considering in our next field work, such as the commercially available magnetic bead-based strategy, non-commercial ROSE extraction method and heated NaOH method. All these considering methods are cheap and easy to perform which will greatly decrease the whole cost.

## Conclusion

In this study, we developed an RPA assay for detection of *S. japonicum* and firstly applied it to epidemiological studies. Our results showed that the RPA assay was a very attractive nucleic acid detection method for the diagnosis of *S. japonicum* infection. This method exhibited high sensitivity, good specificity, convenient operation, minimal equipment requirement and rapid detection. And it could be applicable to not only schistosomiasis diagnosis but also environmental monitoring.

## Abbreviations

ELISA: Enzyme-linked immunosorbent assay
IHA: Indirect hemagglutination assay
KK: Kato-Katz thick smear
MHT: The miracidium hatching test
NPV: Negative predictive value
PPV: Positive predictive value
RPA: Recombinase polymerase amplification
